# The role of artificial intelligence in the management of liver diseases

**DOI:** 10.1002/kjm2.12901

**Published:** 2024-10-23

**Authors:** Ming‐Ying Lu, Wan‐Long Chuang, Ming‐Lung Yu

**Affiliations:** ^1^ Division of Hepatobiliary, Department of Internal Medicine, Kaohsiung Medical University Hospital Kaohsiung Medical University Kaohsiung Taiwan; ^2^ School of Medicine and Hepatitis Research Center, College of Medicine and Center for Liquid Biopsy and Cohort Research Kaohsiung Medical University Kaohsiung Taiwan; ^3^ School of Medicine and Doctoral Program of Clinical and Experimental Medicine, College of Medicine and Center of Excellence for Metabolic Associated Fatty Liver Disease National Sun Yat‐sen University Kaohsiung Taiwan

**Keywords:** algorithms, artificial intelligence (AI), hepatitis C virus (HCV), hepatocellular carcinoma (HCC), machine learning (ML)

## Abstract

Universal neonatal hepatitis B virus (HBV) vaccination and the advent of direct‐acting antivirals (DAA) against hepatitis C virus (HCV) have reshaped the epidemiology of chronic liver diseases. However, some aspects of the management of chronic liver diseases remain unresolved. Nucleotide analogs can achieve sustained HBV DNA suppression but rarely lead to a functional cure. Despite the high efficacy of DAAs, successful antiviral therapy does not eliminate the risk of hepatocellular carcinoma (HCC), highlighted the need for cost‐effective identification of high‐risk populations for HCC surveillance and tailored HCC treatment strategies for these populations. The accessibility of high‐throughput genomic data has accelerated the development of precision medicine, and the emergence of artificial intelligence (AI) has led to a new era of precision medicine. AI can learn from complex, non‐linear data and identify hidden patterns within real‐world datasets. The combination of AI and multi‐omics approaches can facilitate disease diagnosis, biomarker discovery, and the prediction of treatment efficacy and prognosis. AI algorithms have been implemented in various aspects, including non‐invasive tests, predictive models, image diagnosis, and the interpretation of histopathology findings. AI can support clinicians in decision‐making, alleviate clinical burdens, and curtail healthcare expenses. In this review, we introduce the fundamental concepts of machine learning and review the role of AI in the management of chronic liver diseases.

## 1 INTRODUCTION

Chronic liver disease represents a substantial health burden for society. Risk factors for chronic liver diseases include hepatitis B virus (HBV) and hepatitis C virus (HCV) infections, alcohol consumption, and steatotic liver disease.^1^ Regardless of the etiology, chronic liver disease is characterized by chronic inflammation, hepatic fibrogenesis, and progression to hepatocellular carcinoma (HCC). The changes in the etiological factors and therapeutic paradigm for chronic liver disease, encompassing the growing coverage of universal neonatal HBV vaccination, increasing use of high‐efficacy direct‐acting antivirals (DAA) for HCV, increasing obesity, and escalating alcohol abuse, are reshaping the epidemiology of liver diseases.^2^

Despite these changes, adequate management of chronic liver diseases remains an unmet need. Moreover, mass screening of undiagnosed HBV/HCV patients remains an unsolved issue on the path to achieving the World Health Organization (WHO) goal of HBV/HCV elimination. Nucleotide analogs can yield sustained HBV DNA suppression but rarely lead to a functional cure (i.e., HBsAg seroclearance). Moreover, the complexity and lack of consensus in current HBV treatment guidelines may hinder clinical practice, especially for patients in the “gray zone”.^3^ Although the treatment efficacy of DAA is as high as 98%,^4^ it cannot eliminate the risk of HCC and liver‐related complications.^5^ In this regard, cost‐effective identification of high‐risk populations that require HCC surveillance after achieving a sustained virological response is an important concern, as is the construction of a precision medicine‐guided strategy incorporating clinical and molecular biomarkers for HCC therapy.^6^, ^7^ Thus, many challenges in the diagnosis and management of chronic liver diseases are currently unresolved.

**FIGURE 1:**
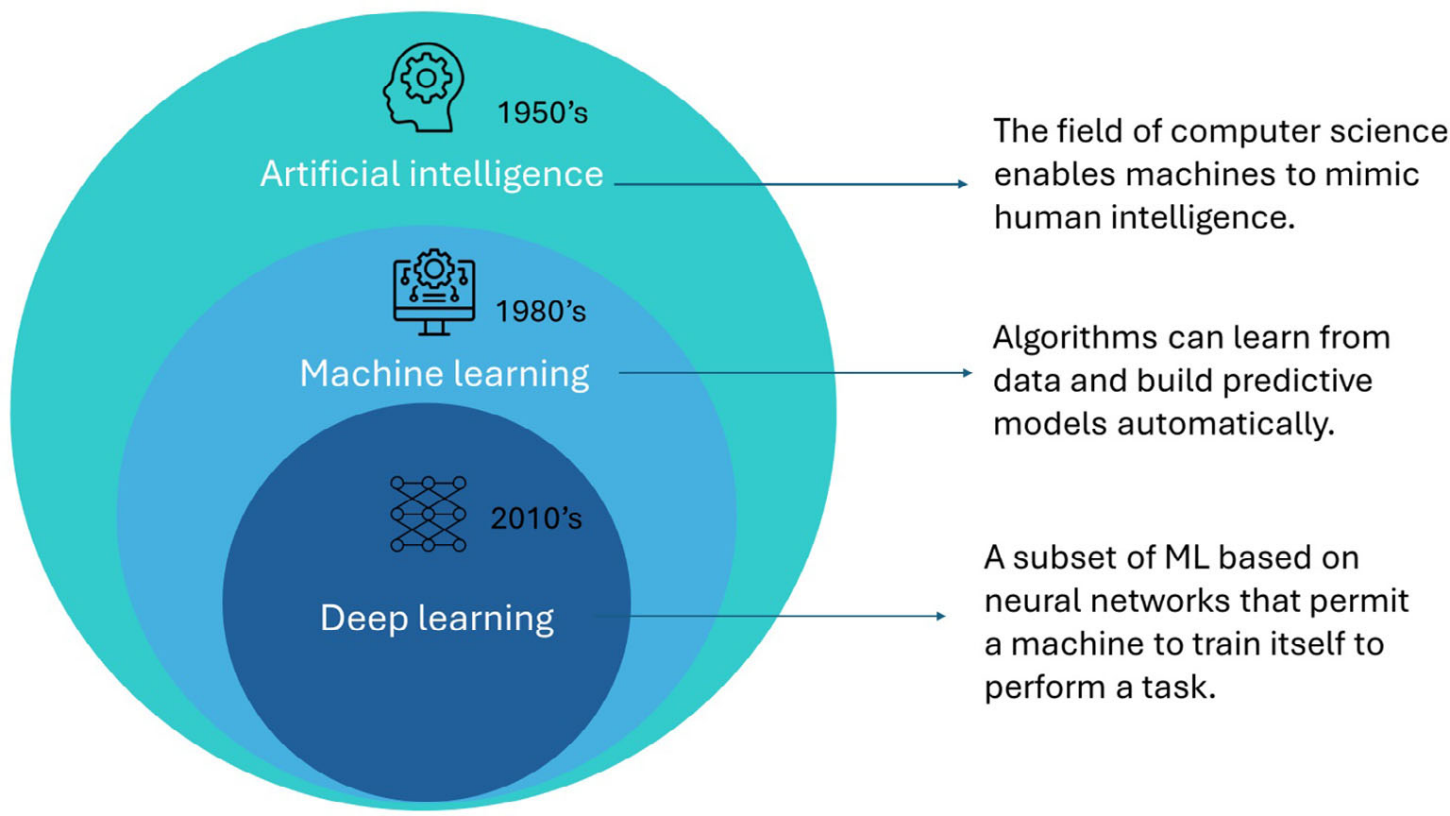
Definitions of artificial intelligence (AI), machine learning (ML), and deep learning (DL).

Advancements in artificial intelligence (AI) have created a new era in healthcare and clinical medicine. The term AI refers to computer software that mimics human cognitive functions such as learning and problem solving. Machine‐learning (ML) is a branch of AI used for data mining and the development of descriptive or predictive models. Deep learning utilizes artificial neural networks to automatically learn and extract the underlying patterns from datasets (Figure 1). These algorithms can handle complex, non‐linear clinical data and identify their underlying relationships, which are difficult to analyze using traditional statistics. The existing applications of AI have focused on risk stratification, outcome prediction, and imaging diagnosis. In this article, we introduce how AI processes data to establish a predictive model and review the role of AI in the management of chronic liver diseases.

## 2 DATA PROCESSING

**FIGURE 2:**
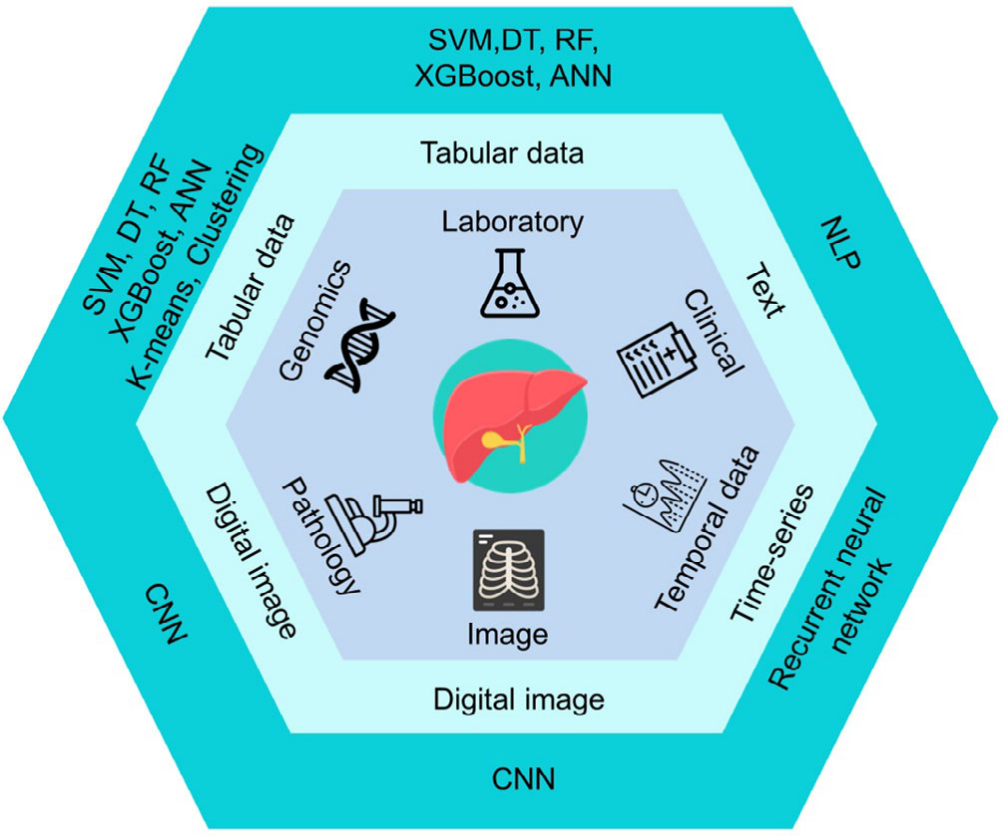
Data types in machine learning analysis. Most supervised machine‐learning approaches are typically used for labeled structured tabular data. Convolutional neural networks (CNNs) are effective for handling digital images. Recurrent neural networks (RNNs) are well‐suited for analyzing time‐series data. Natural language processing (NLP) methods are necessary to extract and process unstructured medical text. Multimodal integrated analysis is essential for the advancement of precision medicine.

**FIGURE 3:**
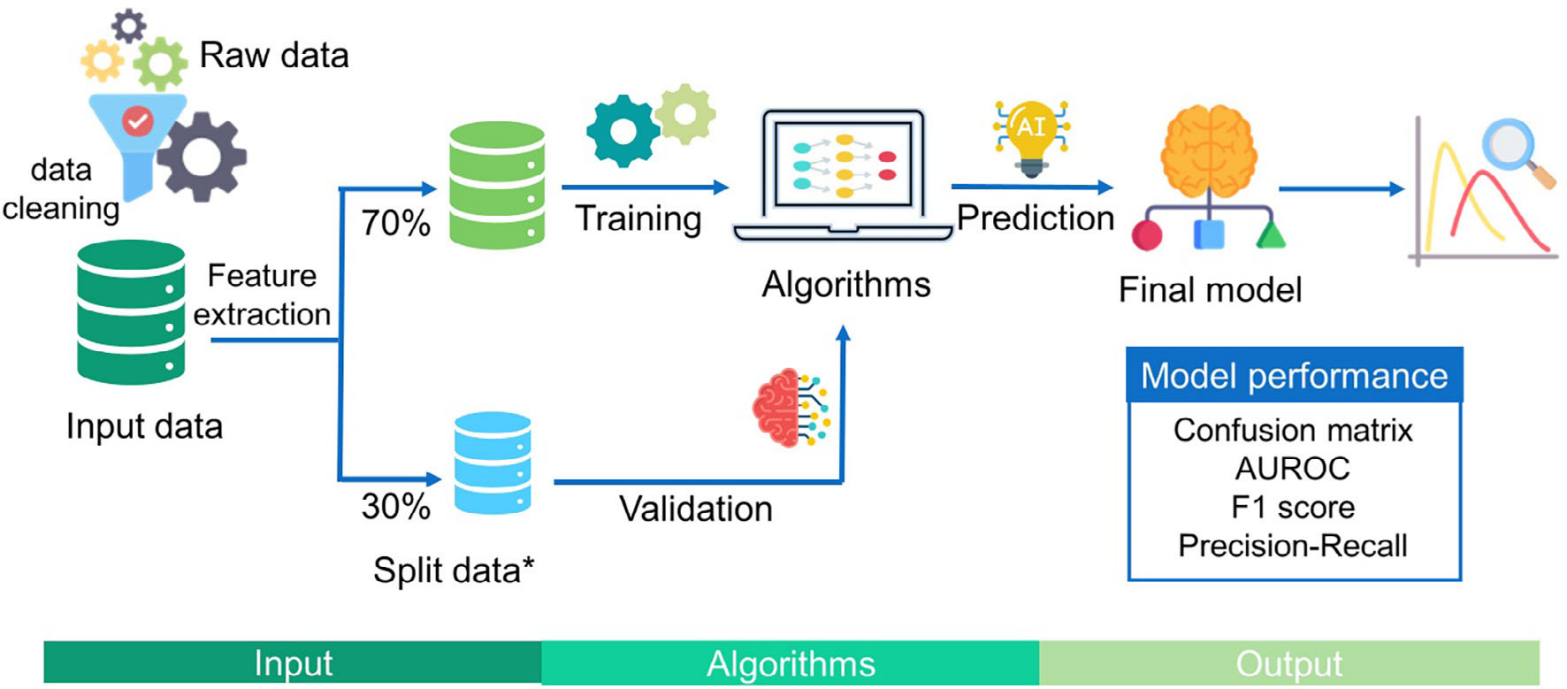
Steps in supervised machine learning. Machine learning begins with input raw data, followed by data preprocessing, feature extraction, algorithm selection, model training, validation, and deployment. The performance of the model can be evaluated using various metrics such as the confusion matrix, AUROC curve, precision‐recall curve, and F1 score. *The training/test dataset ratio can be adjusted, typically from 60%/40% to 90%/10%.

High‐quality data form the foundation of ML models. Raw data are collected and cleaned in the preprocessing phase to remove noise, standardize the data, and reduce dimensionality. After transforming the data into an appropriate format, algorithms are chosen on the basis of the data structure. Figure 2 illustrates the algorithms corresponding to various data structures.^8^ Programming tools such as Python and R Studio offer robust platforms for training ML models. They provide a wide range of libraries and frameworks that make it easier to efficiently build, test, and deploy models. The input data are further divided into training and validation datasets. Algorithms are used to establish predictive models for the input data. Feature extraction methods include univariate selection, correlation matrix, feature importance, and recursive feature elimination or addition.^9^ Evaluation of AI models allow assessment of their performance in solving specific issues. Model optimization can be accomplished by hyperparameter tuning for various configurations^10^ (Figure 3).

## 3 MACHINE LEARNING ALGORITHMS

**FIGURE 4:**
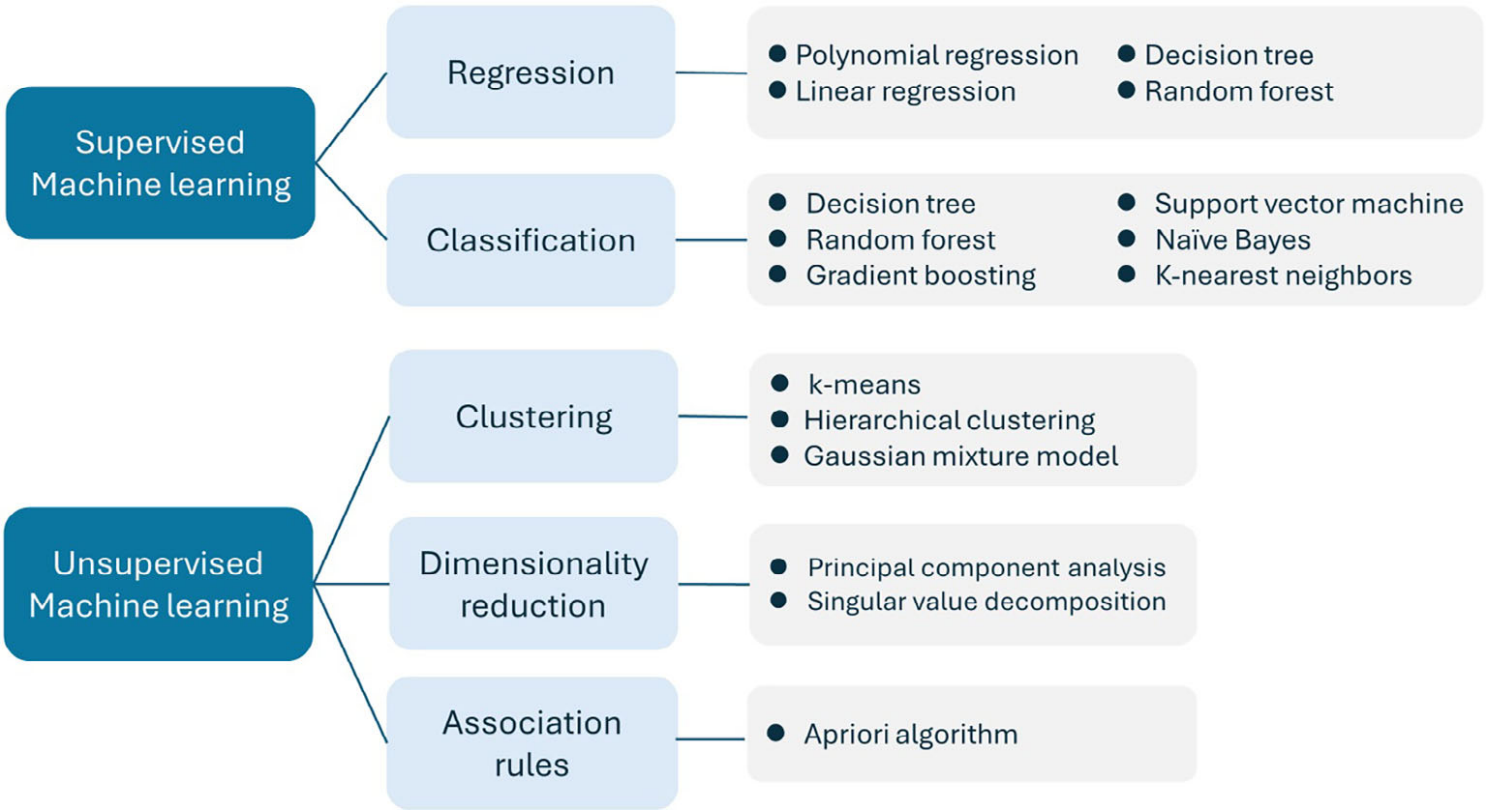
The classification of machine‐learning algorithms.

Machine learning algorithms can be categorized into supervised and unsupervised learning algorithms (Figure 4). Supervised learning algorithms use labeled datasets to train models for prediction. Supervised learning can be further divided into classification and regression tasks. In contrast, unsupervised learning uncovers hidden patterns within unlabeled data. These algorithms process complex unlabeled datasets to find meaningful clusters (k‐means or hierarchical clustering) or reduce dimensionality (principal component analysis).^11^, ^12^ Unsupervised ML can complement supervised ML methods by filtering important features prior to model development.^13^ Since most ML applications in hepatology involve supervised algorithms, we have focused on such approaches.

### 3.1 Decision tree analysis

A decision tree (DT) is a supervised ML algorithm used for classification and regression tasks. It has a hierarchical tree structure composed of root, branch, internal and leaf nodes. The information gain represents the decrease in entropy, while the highest information gain indicates the best attribute for splitting a dataset at each node. The Gini index measures the probability of misclassifying randomly selected samples at a specific node. It calculates the corresponding threshold for splitting the input data into subbranches. The Gini index ranges from 0 to 1, with 0 indicating that all the elements belong to the same category and 1 signifying that all the elements are randomly dispersed across different categories. This splitting process is repeated in a top‐down recursive manner until maximal homogeneity is achieved. Decision trees facilitate the understanding of the most important attributes.^14^

### 3.2 Support vector machines

Support vector machines (SVMs) search for a hyperplane (i.e., decision boundary) in an n‐dimensional space to categorize distinct classes. The optimal hyperplane is determined by calculating the appropriate curvature of the plane and maximizing the distances among various classes. SVMs possess several built‐in kernel functions to separate non‐linear data, including polynomial, sigmoid, and radial basis functions.^15^, ^16^

### 3.3 Random forest

A random forest (RF) is an ensemble ML approach used for regression and classification tasks. The ensemble technique combines multiple weak classifiers to obtain a better predictive performance. The random forest builds multiple decision trees via bootstrapping and combines their predictions to make a final decision by majority voting (classification task) or averaging (regression task). The random forest approach involves variability among individual trees, diminishing overfitting and enhancing predictive accuracy.^17^

### 3.4 eXtreme gradient boosting

The eXtreme gradient boosting (XGBoost) technique is an ensemble technique that uses a gradient‐boosting framework. It is known for its high speed, efficiency, and scalability. XGBoost builds upon an ensemble of decision trees and sequentially adds new trees to correct the errors made by previous models.^18^ It employs a gradient descent method to minimize loss when creating new models.^19^ Theoretically, each new model fits new observations more precisely, thus improving overall accuracy.^20^

### 3.5 Artificial neural networks

An artificial neural network (ANN) consists of an input layer, multiple hidden layers, and an output layer, where the nodes are interconnected. In the hidden layer, each node receives inputs from the preceding layer and computes the corresponding weights and thresholds. If the output of a node exceeds a threshold, the node is activated and transmits information to the next layer. Otherwise, it does not send any data forward. During the training process, the model performance is enhanced by optimizing the prediction from the previous layer through weight adjustment for each feature.^21^

### 3.6 Convolutional neural networks

**FIGURE 5:**
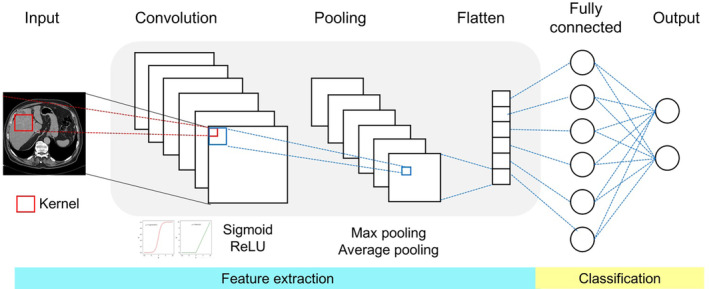
Basic CNN architecture. The basic architecture of a CNN consists of convolutional, pooling, and fully connected layers. The convolutional layers apply kernels to transform images into feature maps. The pooling layers reduce the dimensionality of the feature maps, thereby streamlining the computational process. The flatten layer converts the pooled feature maps into a single vector. The fully connected layer creates interconnected networks and assigns corresponding weights to each neuron. Finally, these features are used to classify the input images into designated output categories.

A convolutional neural network (CNN) is a specialized deep‐learning algorithm for image detection, classification, and segmentation.^22^ Image processing in a CNN includes several steps: The convolution layer applies filters to extract features autonomously. This layer consists of numerous small square templates (i.e., kernels) that slide across an image to detect patterns. If a part of the image matches the kernel pattern, it yields a high positive value. Otherwise, it returns zero or a lower value. Activation functions, such as sigmoid and rectified linear unit (ReLU) functions, introduce non‐linearity into the convolution layer. The convolution layer can be condensed into a single matrix multiplication (i.e., feature map). The pooling layer reduces the dimensionality of the feature maps, preserving essential information while reducing computational load. The flatten layer converts the pooled feature maps into a single vector, which can be input into the fully connected layers for classification tasks^23^ (Figure 5).

### 3.7 Recurrent neural networks

A recurrent neural network (RNN) is a type of neural network that utilizes feedback loops to process data sequences and influence the final output. Therefore, RNN models can handle sequential data and predict subsequent data points. RNNs are commonly applied in scenarios involving language modeling or time‐series data analysis.^24^

### 3.8 Natural language processing

Natural language processing (NLP) applies neural network algorithms to automatically extract, classify, and analyze useful features from unstructured data such as text and voice. Tokenization is the process of splitting a sequence of texts into smaller units as tokens. These tokens can be words, phrases, or even characters, which allows the computer to treat each instance as a distinct value. This process also includes the removal of stop words with no significant meaning. Other NLP tasks include stemming (reducing words to root forms) and part‐of‐speech tagging (assigning grammatical roles to each word in a sentence).^25^ Incorporating NLP to extract information from electronic health records enhances the identification of disease‐related variables in cases lacking International Classification of Diseases (ICD) codes. This markedly reduces the burden of paperwork and improves reporting quality metrics.^26^

## 4 EVALUATION OF MODEL PERFORMANCE

The choice of the metric depends on the task and nature of the data. For classification tasks, common metrics include the confusion matrix, area under the receiver operating characteristic curve (AUROC), precision, recall, and F1 score. The confusion matrix shows the sensitivity, specificity, and positive/negative predictive values. The overall performance can be summarized by the AUROC, which illustrates the correlation between true‐positive and false‐positive rates at various thresholds. The precision–recall curve is more suitable for imbalanced data. Precision is the ratio of true positives to all positives, while recall is the ratio of true positives to true positives plus false negatives. The F1‐score is the harmonic mean between precision and recall. For regression tasks, the mean squared error (MSE) is a fundamental metric that evaluates the average squared difference between actual and predicted values. A lower MSE signifies greater accuracy in predictions.^27^

## 5 CLINICAL APPLICATIONS

The clinical applications of AI in hepatology include lesion detection, risk stratification, and prediction of treatment efficacy and prognosis. AI can recognize the underlying details of images that are imperceptible to humans, thereby making automatic diagnoses faster and more accurate. AI can also enhance the assessment of liver fibrosis, cirrhosis, and benign or malignant liver tumors. Digital pathology has the potential to predict treatment response and capture more prognostic information from liver biopsies. Integrating clinical and multi‐omics data can strengthen precision medicine for specific subtypes of liver diseases.^28^ We have provided an example using HCV to illustrate the application of AI in clinical practice. Table 1 summarizes the state‐of‐the‐art AI technologies for the management of HCV infection.

**TABLE 1:** Studies of AI in chronic hepatitis C.

Study	Algorithm	Training (*n*)	Validation (*n*)	Modality	Classification	Performance
Non‐invasive test						
Doyle et al. 2020^29^	Logistic regression Random forests Gradient boosting Stacked ensemble	7,777,538	972,192	Longitudinal medical history Prescription data	Screening for undiagnosed HCV patients	Validation AUC = 0.96 PPV = 97% Sensitivity = 50% Specificity = 99%
Hashem et al. 2018^30^	Decision tree Genetic algorithm Particle Swarm Optimization Multi‐linear regression	22,690	16,877	Clinical data Serum biomarkers	Advanced liver fibrosis F0–2 versus F3–4	AUC = 0.73–0.76 Accuracy = 66.3%–84.4%
Konerman et al. 2015^32^	Random forest Boosting	533	183	Clinical data Laboratory Histology	Fibrosis progression Liver related outcome	Fibrosis progression AUC = 0.79 Clinical progression AUC = 0.86
Outcome						
Lu et al. 2024^33^	Decision tree Random forest XGBoost Artificial neural network	23,955	10,346	Sociodemographic clinical data Virologic data	Directing‐acting antivirals failure	Training AUC = 1.000 Validation AUC = 0.803
Park et al. 2022^57^	Elastic net Random forest Gradient Boosting Forward neural network	4894	1631	Sociodemographic clinical data Virologic data	Directing‐acting antivirals failure	Validation C‐index = 0.64–0.69
Yu et al. 2022^35^	Decision tree Random forest	82	—	Clinical data Circulating angiogenic factors	The efficacy of lenvatinib for unresectable HCC	Tumor response AUC = 0.906 Overall survival AUC = 0.873
Abajian et al. 2018^36^	Logistic regression Random forest	36	Cross validation	Clinical data MRI	Treatment response of TACE for HCC	Accuracy = 78% Sensitivity = 62.5% Specificity = 82.1%, PPV = 50.0% NPV = 88.5%
Lu et al. 2023^37^	Decision tree Random forest	55	—	Clinical data Genomic data	HCC	AUC = 0.913; Accuracy = 95.7% Nomogram AUC = 0.950
Ioannou et al. 2020^38^	Recurrent neural network	48,151	5‐fold cross validation	Clinical data	HCC	AUC = 0.759
Audureau et al. 2020^58^	Decision tree Random survival forest	836	668	Clinical data Fibroscan	HCC	Validation C‐index = 0.70–0.71
Radiology						
Yasaka et al. 2018^39^	Convolutional neural network	534	100	MRI	Staging of liver fibrosis	Validation AUC F2 = 0.85 F3 = 0.84 F4 = 0.84
Sato et al. 2022^40^	Convolutional neural network	864	108	Ultrasound Clinical data	HCC	Validation AUC Ultrasound = 0.721 Ultrasound + clinical data = 0.994
Kim et al. 2021^41^	Convolutional neural network	568	589	CT	HCC	Validation Sensitivity = 84.8% 4.80 false positive per CT scan
Kim et al. 2020^42^	Fine‐tuned convolutional neural network	549 (54,900 images)	54 (4537 images)	MRI	HCC	Training AUC = 0.965 Sensitivity = 94%, specificity = 99% Validation AUC = 0.900 Sensitivity = 87%, specificity = 93%
Yasaka et al. 2018^43^	Convolutional neural network	460 (55,536 images)	100 (100 images)	CT	HCC Other malignant liver tumors Hemangioma Other benign liver tumor Liver cyst Indeterminate liver nodules	Benign versus malignancy Validation AUC = 0.92 Accuracy = 0.84
Pathology						
Cheng et al. 2022^44^	Convolutional neural network	462 (649 WSI)	234 (422 WSI)	Whole slide image	HCC Pre‐malignant Benign liver nodules	Validation AUC = 0.935
Chen et al. 2022^46^	Convolutional neural network	350 (2917 WSI)	120 (504 WSI)	Whole slide image	Microvascular invasion in HCC	Training AUC = 0.904 Validation AUC = 0.871
Saillard et al. 2020^47^	Convolutional neural network	194 (390 WSI)	328 (342 WSI)	Whole slide image	Predicting survival after HCC resection	Training c‐index = 0.75–0.78 Validation c‐index = 0.68–0.70
Zeng et al. 2022^51^	Patch‐based deep learning Classic MIL CLAM	336 (349 WSI)	139 (139 WSI)	Whole slide image RNAseq	Predict the activation of 6 immune gene signatures from histology	CLAM Training AUC = 0.780–0.914 Validation AUC = 0.810–0.921

Abbreviations: CLAM, clustering‐constrained attention multiple‐instance learning; CT, computed tomography; HCV, hepatitis C virus; HCC, hepatocellular carcinoma; MIL, multiple‐instance learning; MRI, magnetic resonance imaging; NPV, negative predictive value; PPV, positive predictive value; TACE, trans‐arterial chemoembolization; XGBoost, eXtreme Gradient Boosting; WSI, whole slide image.

### 5.1 Non‐invasive diagnostic tests

ML facilitates risk stratification for intensive monitoring and management. Identifying undiagnosed patients is a significant challenge for HCV elimination. Doyle et al. applied AI to capture longitudinal medical records linked to prescription data. For recall levels >50%, the ensemble algorithm achieved a positive predictive rate of 97% for screening undiagnosed patients with HCV, which was higher than the HCV prevalence of 2.23% in the general population.^29^ Non‐invasive tests can serve as surrogates for fibrosis staging and potentially avoid liver biopsy. By combining clinical information and serum biomarker data, AI models could discriminate fibrosis stages in a large HCV cohort with accuracy ranging between 66.3% and 84.4%.^30^ Shousha et al. developed an ML model incorporating the IL28B genotype and biochemical parameters to predict advanced fibrosis in patients with HCV, achieving better performance than the fibrosis‐4 index and aspartate aminotransferase‐to‐platelet ratio index.^31^ Konerman et al. constructed AI models to analyze the non‐linear relationships between longitudinal data and fibrosis progression. The random forest model achieved an AUROC of 0.79 for predicting fibrosis progression and 0.86 for liver‐related outcomes.^32^

### 5.2 Outcome prediction

ML facilitates the assessment of treatment response and prognosis of HCV/HCC. Lu et al. conducted a nationwide study (*n* = 34,301) to investigate the risk factors of DAA failure using ML algorithms. The XGBoost model, which consisted of 55 host and virological features, outperformed other algorithms in predicting DAA failure, achieving an AUROC of 1.000 in the training dataset and 0.803 in the validation dataset.^33^, ^34^ Tyrosine kinase inhibitors and immune checkpoint inhibitors are the emerging therapeutic agents for advanced HCC. Hsu et al. employed a decision‐tree‐based survival model that incorporated serum biomarkers, such as the α‐fetoprotein level, albumin‐bilirubin grade, and the levels of angiogenic cytokines, to evaluate the response to lenvatinib in patients with unresectable HCC.^35^ By combining clinical data and baseline magnetic resonance imaging (MRI) features, the random forest model could predict the outcome of transarterial chemoembolization for HCC before the procedure (accuracy = 78%).^36^

AI can also optimize HCC surveillance strategies by refining the risk stratification for patients with HCV. By integrating clinical and genetic biomarkers, Lu et al. applied a decision‐tree algorithm to stratify the risk of HCC among patients with HCV infection following viral eradication. They also created a nomogram to predict the probability of HCC and achieved an AUROC of up to 0.950.^37^ Ioannou et al. trained an RNN model incorporating four baseline and 27 longitudinal clinical variables to predict the occurrence of HCC in a large cohort of patients with HCV‐related cirrhosis (*n* = 48,151). They found that 80% of patients developed HCC within 3 years if the RNN‐derived score exceeded the top 51% (AUROC = 0.759).^38^

### 5.3 Radiology

Applications of deep learning in radiology include staging of hepatic fibrosis, localization of HCC, and differentiation of liver nodules. CNNs have exhibited promising performance in the assessment of liver fibrosis, showing a high correlation between the image and biopsy results. A CNN model extracted from MRI scans could discriminate fibrosis stages, achieving an AUROC of 0.84 for F4, 0.84 for F3, and 0.85 for F2.^39^ A multimodal CNN model incorporating demographics, biochemistry, and ultrasound images outperformed the ultrasound‐only model, boosting the diagnostic accuracy for HCC from 68.5% to 96.3%.^40^ Kim et al. developed a CNN‐based HCC model that exhibited a sensitivity of 84.8% with a false‐positive rate of 4.80% per CT scan.^41^ This technique was applied to detect early‐stage HCC in an MRI series, and it achieved 87% sensitivity and 93% specificity in the validation dataset.^42^ Yasaka et al. utilized a CNN for differentiation of benign and malignant liver nodules on dynamic CT scans and obtained AUROC values up to 0.92.^43^ The accuracy of the CNN models was comparable to that of less‐experienced radiologists.

### 5.4 Histopathology

Liver biopsy is invasive, and its accuracy may be limited by sampling errors and interobserver variability. Digital pathology provides rapid, reliable, reproducible, and quantitative diagnosis of biopsies. AI‐assisted techniques are promising for detecting liver fibrosis, HCC, and microvascular invasion, allowing for risk factor modification and the initiation of appropriate therapy. Although AI‐assisted tools cannot replace pathologists, they have the potential to improve diagnostic accuracy and streamline workflow efficiency.

Liver nodules constitute a group of heterogeneous disorders with distinct clinical implications. Seven subtypes of liver nodules can be distinguished by CNN (AUROC = 0.935), improving risk stratification among patients with early HCC, premalignant or benign nodules.^44^ Microvascular invasion (MVI) is an important predictor of HCC prognosis.^45^ Nevertheless, over half of the patients with HCC lack sufficient peritumoral tissue, complicating the diagnosis of MVI. Chen et al. established an AI model for MVI evaluation with high accuracy (AUROC = 0.87), which showed similar performance even if the patient had only one biopsy specimen or a single section (AUROC = 0.88).^46^ Risk assessment of patients with HCC is crucial for determining therapeutic strategies after curative resection or ablation. Saillard et al. developed CNN models using digital slides to predict the survival of patients with HCC after surgical resection; the models surpassed the accuracy of traditional clinical, biological, and pathological predictors.^47^

Immune checkpoint inhibitors represent a therapeutic breakthrough for advanced HCC. However, anti‐PD1 inhibitors only show outstanding response rates in a small fraction (20%–35%) of patients.^48^ HCC patients overexpressing immune gene signatures have increased sensitivity to anti‐PD1 therapy.^49^, ^50^ AI‐based pathological assessments can predict six panels of distinct gene signatures linked to the efficacy of anti‐PD1 inhibitors directly from histological findings.^51^ The molecular classification of HCC is primarily based on the intrinsic features of cancer cells, which may disregard the role of the microenvironment in carcinogenesis.^52^ Wang et al. developed a CNN model for automated single‐cell segmentation and classification of digital slides. This technology quantifies individual cells and extracts spatial relationships between cancer cells and infiltrating lymphocytes. Using unsupervised clustering analysis, the three HCC subtypes were found to be linked to specific genomic alterations and molecular pathways. These subtypes exhibit prognostic significance beyond the conventional clinicopathologic factors.^53^

## 6 CHALLENGES AND FUTURE PERSPECTIVES

Although AI technology is beginning to flourish in hepatology, its applications in this field remain nascent and face numerous challenges. The limitations of AI models include overfitting, poor generalizability, and limited data explainability. The predictive power and reproducibility of AI models are highly dependent on the quantity and quality of input data. Most AI models are considered “black boxes” that are too complex to be understood by humans. Transparency is crucial for clinicians to realize and trust the predictions made by AI models. Explainable AI, based on the Shapley method, can overcome this important issue in clinical practice.^54^ Shapley additive explanations (SHAP) provide a global view of feature importance by quantifying the contribution of each variable to the outcome.^10^ The accessibility of high‐throughput next‐generation sequencing (NGS) has accelerated the advances in precision medicine. By integrating AI with multi‐omics, clinicians can tailor individual diagnostic and treatment strategies, moving away from the “one‐size‐fits‐all” approach. High‐quality data, novel biomarker discovery, and algorithms are the primary requirements for enhancing the performance of AI models.

A wide gap exists between the growing body of research on AI and its limited real‐world clinical applications. This discrepancy may result from a lack of external validation, insufficient technological infrastructure, limited knowledge among medical personnel, or concerns about data privacy.^55^ The challenge with AI lies in the systematic extraction and integration of information across various modalities. The integration of multimodal data can strengthen the clinical applicability of AI models. High‐quality standardized data are instrumental in improving the generalizability and interpretability of the predictive results. Automated ML (AutoML) offers a user‐friendly framework that makes AI accessible to users without extensive data science expertise. AutoML platforms begin with raw data and yield deployable AI models with verified performance metrics on the validation dataset.^56^ AutoML platforms can serve as solutions to AI challenges, facilitating the implementation of AI in various clinical settings in the near future.

## 7 CONCLUSIONS

AI heralds a new epoch in precision medicine. The availability of high‐throughput genomic data provides opportunities for AI applications in this field. Integrating AI with multi‐omics can contribute to disease diagnosis, novel biomarker discovery, and the prediction of treatment efficacy and prognosis. AI can support clinicians in decision‐making, streamlining clinical workflows, and reducing healthcare expenses.

## CONFLICT OF INTEREST STATEMENT

All authors declare no conflict of interest.
